# Belowground stressors and long-term seagrass declines in a historically degraded seagrass ecosystem after improved water quality

**DOI:** 10.1038/s41598-017-14044-1

**Published:** 2017-10-31

**Authors:** Matthew W. Fraser, Gary A. Kendrick

**Affiliations:** School of Biological Sciences and Oceans Institute, The University of Western Australia, 35 Stirling Highway, Crawley, WA 6009 Australia

## Abstract

Continued seagrass declines in ecosystems with improved water quality may be driven by sediment stressors. One of the most cited examples of a seagrass ecosystem with declines is Cockburn Sound, Western Australia, where 75% of seagrasses (2169 ha) were lost in the 1960s–1980s due to poor water quality. Water quality has subsequently improved in Cockburn Sound, yet shoot density declines continue in some areas. Here, we investigated if sediment stressors (sulfide intrusion and heavy metals) contributed to declining *Posidonia sinuosa* shoot densities in Cockburn Sound. Seagrass δ^34^S were depleted at sites with a history of seagrass declines, indicating seagrasses at these sites were under sulfide stress. Heavy metals (Fe, Zn, Mn, Cr, Cu and Cd) in sediments and seagrasses did not show clear patterns with shoot density or biomass, and largely decreased from similar measurements in the late 1970s. However, seagrass cadmium concentrations were negatively correlated to seagrass biomass and shoot density. High cadmium concentrations interfere with sulfur metabolism in terrestrial plants, but impacts on seagrasses remain to be explored. Given that sulfide intrusion can prevent recolonization and drive seagrass declines, management plans in degraded seagrass ecosystems should include management of sediment stressors and water quality to provide comprehensive management.

## Introduction

Seagrass declines threaten the substantial ecological and economic services that seagrass ecosystems provide. Seagrass loss has historically been linked to reduced water quality driven by disturbances such as coastal development and nutrient inputs^1^. These disturbances reduce light availability through inputs of fine sediments or by allowing the sudden growth of phytoplankton or epiphytic algal blooms. Given that seagrasses require relatively high light concentrations, reduced light availability can lead directly to sudden seagrass declines and dieback events^2,3^. In addition, reduced light availability limits the capacity of plants to oxygenate surrounding sediments through radial oxygen loss from roots^4^, which can increase the susceptibility of seagrasses to sulfide intrusion by limiting the diel cycling of sulfide and iron in the rhizosphere^5^. However, there are several examples of sudden seagrass diebacks in areas where water quality is good^6–8^. This suggests that other environmental factors likely play an equally important role in seagrass declines. Where water quality is improved but seagrass declines continue, belowground stressors likely drive seagrass declines and slow recovery^8^, yet our knowledge of the ecophysiological impacts of such stressors on seagrasses are limited.

The widespread (2169 ha) loss of seagrass in Cockburn Sound between the 1960’s and 1980’s^3^ was primarily attributed to low light availability driven by industrial inputs that lowered water quality, enhanced phytoplankton blooms and increased light attenuation^9^. As such, subsequent management projects have emphasized pressure response models linking seagrass loss with water quality. Since the 1980’s, water quality has considerably improved in Cockburn Sound and, in general, meets all environmental quality guidelines^10^. However, long-term seagrass monitoring shows significant declines in seagrass density at several sites in Cockburn and Warnbro Sounds, despite the improved water quality^11^. The decline in seagrass is patchy across the area, with sites around Garden Island and reference sites in Warnbro Sound showing the greatest rates of decline^12^. The juxtaposition between improved water quality but continued seagrass decline suggests that the current management model must be modified to account for drivers of seagrass loss other than poor water quality.

The intrusion of hydrogen sulfide from sediments has been implicated as a factor in seagrass declines globally^7,8,13^. Sediments are elevated in sulfides relative to seawater, and can be toxic to seagrasses^14^. The susceptibility of seagrasses to sulfide intrusion depends on the capacity for seagrasses to oxygenate the surrounding rhizosphere which can be influenced by physio-chemical conditions in sediments^4^. For example, an increase in sedimentary organic matter (OM) decreases oxygen availability and increases sulfate reduction through enhanced rates of microbial activity, thereby increasing the susceptibility of seagrasses to sulfide intrusion^15,16^. Elevated OM has been hypothesised as a driver of increased sulfide intrusion in temperate WA seagrasses including *Posidonia sinuosa* at Two Peoples Bay and Woodman Point^17^. Conversely, the presence of iron in sediments promotes the formation of iron-sulfide compounds like pyrite, reducing the possibility of sulfide intrusion into seagrass tissues^18,19^. However, the capacity for iron to buffer sulfide concentrations in sediments depends on reoxidation of FeS species formed, which can change appreciably over diel and seasonal periods^5,20^. Understanding differences in the composition and chemical nature of sediments, and how they change over time, will assist in identifying seagrass meadows that may be at greater risk of declines from sulfide intrusion. The isotopic ratio of sulfur (δ^34^S) can be used as an indicator for intrusion of sediment sulfides into seagrasses. More negative values indicate a higher contribution of sulfides from sediments (sediment average: −15‰ to −25‰), while a more positive value indicates that sulfate from the water column is the dominant S source for the plant (seawater average: +21‰)^21^.

Anthropogenic pollutants such as heavy metals have also previously been implicated as drivers of seagrass declines, particular in areas adjacent to coastal development^22^. Seagrasses can take up heavy metals from the water column through leaves, or from sediments through root uptake^23^. After inputs are reduced, tides or other hydrodynamic forces can remove pollutants over relatively short time scales (days-weeks), but heavy metals in sediments can persist for longer (years-decades) where mobility of the pollutants is limited^24^. The mobility of metals in marine sediments often depends on interactions with sulfides and iron, which changes depending on oxygen availability. Cationic heavy metals form solid phase sulfides when sulfide concentrations increase under anoxic conditions, but are remobilized when sediments become oxygenated^25^. Conversely, oxyanions are immobilized through sequestration with Fe(III) under oxygenated conditions, but become mobilised under reduced conditions when Fe(III) minerals are reduced to Fe(II)^26^. As such, metals may impact seagrasses even after inputs have ceased. Following uptake, metals can have varying effects on seagrass physiology and health. For example, seagrasses exposed to Cu and Zn show decreased photosynthetic efficiency^22^, while Cu, Ni, and Cr additions decreased leaf cell viability^27^. Long-term exposure to metals at slightly elevated concentrations may also decrease seagrass health, or lower seagrass resilience to other stressors.

We aimed to determine if sediment stressors (sulfide intrusion and heavy metals) were linked to continuing declines in shoot density of *Posidonia sinuosa* in Cockburn Sound. Specifically, we focused on the potential role of belowground stressors on *P. sinuosa* meadows across the region. We hypothesised that (i) sulfide intrusion from sediments into seagrass leaves (measured by δ^34^S) would be greatest at sites with highest rates of seagrass declines and not related to depth, (ii) seagrass δ^34^S would be positively correlated with sediment OM concentrations, (iii) seagrass δ^34^S would be negatively correlated with sediment iron concentrations and (iv) sites with seagrass declines would have higher concentrations of metals in sediments and seagrass tissues.

## Results

### Seagrass shoot density, biomass, and productivity

*Posidonia sinuosa* shoot density significantly differed across the study locations (F_2,33_ = 4.8, p = 0.015), with Warnbro Sound (WS) having lower densities than Eastern Banks (EB) and Garden Island (GI) (Table 1). Shoot density ranged from a minimum of 300 shoots m^−2^ at WS 3 m to a maximum of 2050 shoots m^−2^ at GI 2 m. Leaf biomass also significantly differed between locations (F_2,33_ = 7.46, p = 0.002), driven by lower biomass at WS sites. Root and rhizome biomass did not significantly differ across study locations, but significantly differed between depths (Roots: F_3,32_ = 6.7, p = 0.001; Rhizomes: F_3,32_ = 3.94, p = 0.017) driven by higher biomass at 2 m sites. There were no significant differences in above:belowground biomass ratios between locations or depths, showing changes in belowground biomass were not a result of a shift in biomass allocation. Productivity (leaf production) did not significantly differ between locations or depths (Table 1).

**Table 1:** Mean seagrass shoot densities, biomass and productivity across Cockburn/Warnbro Sound.

Site	Shoot density (shoots m^−2^)	Leaf biomass (g m^−2^)	Rhizome biomass (g m^−2^)	Root biomass (g m^−2^)	Productivity (mg sh day^−1^)
GI 2 m	2050 (317)	241.3 (32.0)	263.5 (60.2)	182.3 (27.4)	1.03 (0.05)
GI 3 m	1042 (102)	88.9 (24.8)	145.6 (41.7)	49.8 (10.8)	1.37 (0.07)
GI 5 m	1450 (156)	188.0 (19.1)	300.4 (88.0)	102.8 (19.6)	1.43 (0.06)
GI 7 m	858 (245)	104.7 (20.1)	130.8 (48.3)	36.8 (14.4)	1.22 (0.09)
EB 2 m	1042 (158)	94.2 (10.2)	172.8 (41.8)	24.6 (9.8)	1.30 (0.09)
EB 7 m	1425 (300)	133.9 (34.3)	267.0 (46.0)	63.5 (33.7)	1.05 (0.06)
EB 5 m	1567 (82)	162.5 (13.5)	202.5 (54.2)	72.6 (23.7)	1.03 (0.04)
EB 3 m	1758 (1000)	154.5 (90.5)	226.3 (32.1)	84.7 (57.8)	1.07 (0.05)
WS 2 m	1308 (188)	58.0 (13.2)	321.7 (84.0)	72.9 (25.6)	1.16 (0.05)
WS 3 m	300 (66)	24.7 (9.1)	17.3 (6.9)	1.2 (0.8)	1.48 (0.11)
WS 5 m	1100 (292)	105.8 (36.8)	168.5 (42.4)	67.2 (17.2)	0.84 (0.05)
WS 7 m	658 (129)	89.9 (12.3)	120.7 (62.1)	40.0 (9.7)	1.10 (0.05)

GI = Garden Island, WS = Warnbro Sound. Means are calculated from three replicates, with standard errors provided in brackets.

### Seagrass sulfur

**Figure 1:**
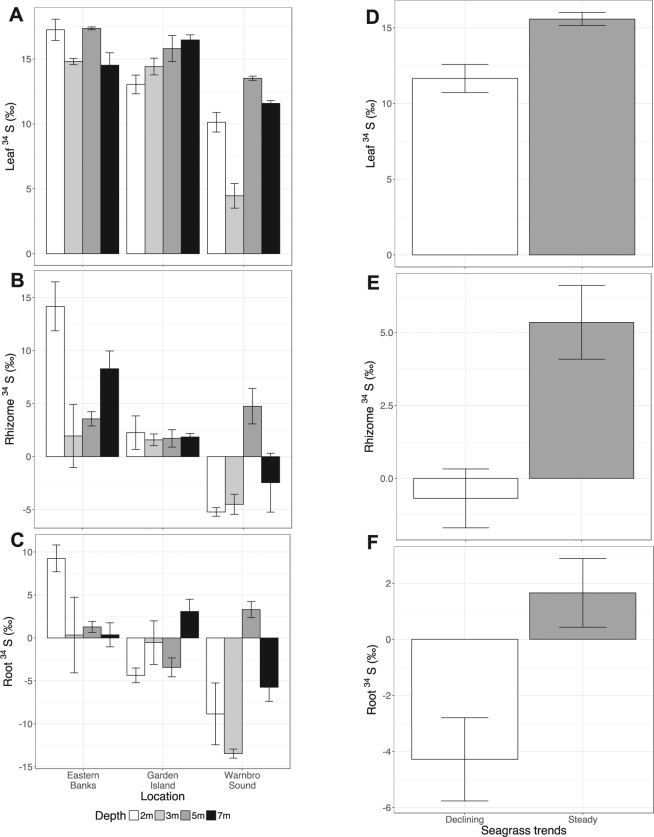
Mean δ^34^S for seagrasses in Cockburn and Warnbro Sound. (**A**) Leaf, (**B**) Rhizome and (**C**) Root δ^34^S of seagrasses across three regions in Cockburn and Warnbro Sound. Colour of bars represent depth of sites (white = 2 m, light grey = 3 m, dark grey = 5 m, black = 7 m). Each bar represents the mean δ^34^S at each site, with error bars representing 1 standard error (n = 3). Mean (**D**) Leaf, (**E**) Rhizome and (**F**) Root δ^34^S grouped by long-term trends in shoot density at each site. White bars represent sites with a history of shoot declines, while grey bars are from sites with no shoot declines^12^. Each bar represents the mean δ^34^S, with error bars representing 1 standard error (n = 18).

Seagrass δ^34^S values showed distinct differences across the study area (Table 2). Leaf δ^34^S significantly differed between Warnbro, Eastern Cockburn and Garden Island Sites (F_2,33_ = 19.8, p < 0.0001; Fig. 1A), but did not differ between depths (F_3,32_ = 2.52, p = 0.076). Rhizome δ^34^S significantly differed between locations (F_2,33_ = 11.9, p = 0.0001; Fig. 1B) but not between depths (F_3,32_ = 19.8, p = 0.434). Similarly, root δ^34^S significantly differed between locations (F_2,33_ = 6.6, p = 0.004; Fig. 1C) but not depths (F_3,32_ = 0.5, p = 0.68). Plants from Warnbro Sound consistently had the most depleted δ^34^S values in leaves, rhizomes, and roots, while Garden Island plants had more depleted δ^34^S signatures than Eastern Banks sites. Leaf δ^34^S was positively correlated to leaf biomass (R^2^ = 0.18, p = 0.005, n = 36), though this relationship was weak. Rhizome δ^34^S and root δ^34^S were not correlated to rhizome or root biomass respectively. Leaf δ^34^S was strongly correlated to rhizome δ^34^S (R^2^ = 0.40, p < 0.0001, n = 36). Rhizome δ^34^S was strongly correlated to root δ^34^S (R^2^ = 0.63 p < 0.0001, n = 36).

**Table 2:** Mean seagrass total sulfur (% d.w.) and sulfur isotope composition (δ^34^S) values in leaves, roots and rhizomes across Cockburn/Warnbro Sound, plus comparison to similar data from other locations. Means are calculated from 3 replicates, with standard errors provided in brackets. *Root biomass was too low at WS 3 m for root S and δ^34^S in one replicate.

Site	Leaf S (%)	Rhizome S (%)	Root S (%)	δ^34^S Leaf (‰)	δ^34^S Rhizome (‰)	δ^34^S Root (‰)
GI 2 m	0.18 (0.009)	0.49 (0.04)	0.76 (0.07)	13.04 (0.7)	2.26 (1.6)	−4.36 (0.9)
GI 3 m	0.18 (0.01)	0.61 (0.02)	0.53 (0.05)	14.42 (0.6)	1.58 (0.5)	−0.54 (2.5)
GI 5 m	0.21 (0.04)	0.53 (0.01)	0.61 (0.05)	15.80 (1.0)	1.72 (0.8)	−3.43 (1.1)
GI 7 m	0.20 (0.02)	0.54 (0.04)	0.54 (0.03)	16.47 (0.4)	1.85 (0.3)	3.08 (1.4)
EB 2 m	0.20 (0.009)	0.52 (0.04)	0.65 (0.1)	14.81 (0.2)	1.95 (3.0)	0.33 (4.4)
EB 7 m	0.17 (0.01)	0.50 (0.01)	0.55 (0.04)	14.52 (1.0)	8.29 (1.7)	0.36 (1.4)
EB 5 m	0.21 (0.01)	0.45 (0.02)	0.68 (0.03)	17.36 (0.1)	3.56 (0.7)	1.28 (0.6)
EB 3 m	0.22 (0.03)	0.31 (0.02)	0.40 (0.02)	17.25 (0.8)	14.18 (2.3)	9.25 (1.6)
WS 2 m	0.17 (0.003)	0.78 (0.08)	0.68 (0.07)	10.13 (0.8)	−5.22 (0.4)	−8.85 (3.6)
WS 3 m	0.20 (0.02)	0.56 (0.04)	1.12*	4.46 (1.0)	−4.51 (0.9)	−13.47*
WS 5 m	0.17 (0.02)	0.49 (0.05)	0.45 (0.01)	13.51 (0.2)	4.76 (1.7)	3.30 (0.9)
WS 7 m	0.20 (0.009)	0.66 (0.1)	0.81 (0.2)	11.58 (0.2)	−2.46 (2.8)	−5.74 (1.6)
SW Aus.^1^	0.88	0.73	0.95	18.3	4.6	0.9
Shark Bay^2^	0.85	0.99	0.78	12.47	−3.17	1.62
Global ave.^3^	0.60	0.60	0.8	12.40	5.10	0.10

^1^Holmer and Kendrick 2013; ^2^Cambridge *et al*. 2012; ^3^Holmer and Hasler-Sheetal 2014.

Sites with a history of significant trends of shoot decline had significantly depleted leaf (F_1,34_ = 14.6, p = 0.0006; Fig. 1D), rhizome (F_1,34_ = 13.9, p = 0.0007; Fig. 1E) and root δ^34^S (F_1,34_ = 9.6, p = 0.004; Fig. 1F) values compared to sites with steady shoot density trends. Leaf, rhizome, and root δ^34^S from Garden Island and Warnbro Sound seagrasses was depleted relative to *P. sinuosa* from south-western Australia^17^, *P. australis* collected in Shark Bay^28^, and global averages for all seagrass species^21^. There was no relationship between sediment OM and δ^34^S in leaves (R^2^ = 0, p = 0.8, n = 36), rhizomes (R^2^ = 0.02, p = 0.4, n = 36), or roots (R^2^ = 0.03, p = 0.28, n = 36).

Leaf sulfur concentrations [S] showed no significant difference between sites, ranging from 0.17% at EB 7 m to 0.22% at EB 3 m (Table 2). Similarly, root [S] was not significantly different between locations or depths, with highest concentrations at WS 3 m (1.12%) and lowest at EB 3 m (0.4%). However, rhizome [S] was significantly higher in sites at Warnbro Sound sites (F_2,33_ = 8.0, p = 0.001), and was highest at WS 2 m (Table 2). Seagrass leaf, rhizome and root [S] in Cockburn/Warnbro Sound tended to be lower than comparable measurements of *P. sinuosa* in other SW Australia estuaries^17^, though root [S] values at WS 3 m were greater.

### Sediment metal concentrations

**Figure 2:**
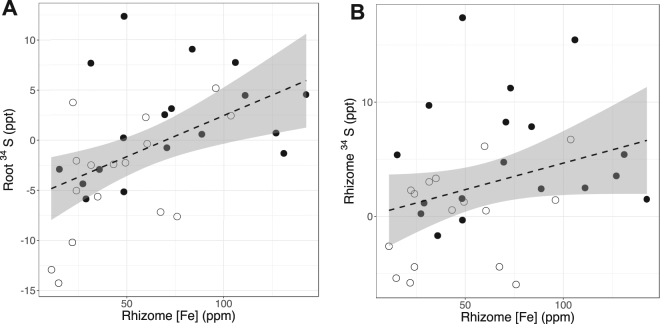
Scatterplots showing relationship between (**A**) rhizome [Fe] and root δ^34^S and (**B**) rhizome [Fe] and rhizome δ^34^S. Dashed lines represent linear model, while grey outline shows 95% confidence intervals. White dots denote replicates from sites with a history of seagrass declines, while black dots denote replicates from sites with steady seagrass trends^12^.

Sediment metal concentrations showed varying patterns between locations (Table 3). Sediment [Cd] was rarely above detectable levels, and showed no significant differences between locations. Sediment [Fe] was not significantly different between sites, but was significantly higher at deeper sites (F_3,32_ = 16.9, p < 0.0001). Sediment [Mn] was also significantly higher at deeper sites (F_3,32_ = 5.2, p = 0.005). Sediment [Cr] was significantly different between locations (F_2,33_ = 11.8, p = 0.0001), with highest values at Warnbro Sound sites. Sediment [Cr] was also greater at shallow sites (F_3,32_ = 7.1, p = 0.001). Sediment [Zn] concentrations were significantly higher in EB Sites, with concentrations above 5 ppm in EB 7 m, EB 5 m, and EB 3 m, and <2 ppm at all other sites. No sediment metal concentrations were significantly correlated to seagrass shoot density (all p > 0.05, n = 36). Root δ^34^S values across Warnbro/Cockburn Sounds was positively correlated to Fe concentrations in sediments (R^2^ = 0.14, p = 0.01, Fig. 2A), while rhizome δ^34^S values were positively correlated to rhizome [Fe] (R^2^ = 0.13, p = 0.04, n = 36, Fig. 2B).

**Table 3:** Mean metal concentrations in sediments in Cockburn/Warnbro Sounds, plus comparable data collected from Warnbro Sound in 1977. Means are calculated from 3 replicates, with standard errors provided in brackets.

Site	Sediment Fe (ppm)	Sediment Zn (ppm)	Sediment Mn (ppm)	Sediment Cr (ppm)	Sediment Cu (ppm)	Sediment Cd (ppm)
GI 2 m	39.37 (1.3)	1.62 (0.6)	1.28 (0.01)	0.92 (0.04)	0.20 (0.01)	0.004 (0.001)
GI 3 m	62.23 (3.0)	1.52 (0.3)	1.46 (0.1)	0.57 (0.01)	0.43 (0.01)	0.008 (0.002)
GI 5 m	51.13 (2.1)	1.25 (0.2)	1.07 (0.03)	0.69 (0.07)	0.24 (0.02)	0.005 (0.001)
GI 7 m	79.43 (9.2)	1.90 (0.3)	1.28 (0.05)	0.60 (0.05)	0.35 (0.03)	0.005 (0.0003)
EB 2 m	34.80 (2.7)	1.66 (0.07)	0.87 (0.02)	0.34 (0.006)	0.24 (0.02)	0.003 (0.001)
EB 7 m	109.73 (3.7)	6.78 (0.9)	2.16 (0.03)	0.27 (0.006)	0.53 (0.01)	0.008 (0.001)
EB 5 m	64.80 (14.1)	7.51 (2.3)	1.14 (0.2)	0.47 (0.009)	0.39 (0.1)	0.002 (0.001)
EB 3 m	57.17 (0.8)	5.51 (0.7)	1.10 (0.01)	0.67 (0.007)	0.41 (0.06)	0.006 (0.002)
WS 2 m	31.70 (3.2)	1.09 (0.8)	1.23 (0.03)	1.01 (0.02)	0.13 (0.003)	0.003 (0.001)
WS 3 m	43.90 (5.5)	0.48 (0.09)	1.36 (0.05)	0.94 (0.02)	0.16 (0.007)	0.004 (0.001)
WS 5 m	83.20 (15.3)	1.29 (0.5)	1.90 (0.1)	0.60 (0.07)	0.41 (0.2)	0.009 (0.002)
WS 7 m	88.83 (3.9)	1.14 (0.3)	1.89 (0.09)	0.57 (0.03)	0.27 (0.02)	0.006 (0.001)
*Cockburn Sound 1977* ^1^	*3647.62*	*15.02*	*10.16*	*25.01*	*11.36*	*0.37*

^1^Talbot and Chegwidden (1983).

### Seagrass metal content

Rhizome [Zn] was significantly higher at sites adjacent to Garden Island (F_3,32_ = 7.1, p = 0.002), primarily driven by high concentrations at GI 2 m and GI 7 m (Table 4). Rhizome [Fe] was not significantly different between locations (F_2,33_ = 2.8, p = 0.07), but was significantly greater at deeper sites (F_3,32_ = 6.7, p = 0.001) due to a positive correlation with sediment [Fe] (R^2^ = 0.27, p = 0.001, n = 36). Sediment [Fe] was weakly positively correlated with root δ^34^S (R^2^ = 0.17, p = 0.015) and rhizome [Fe] was weakly positively correlated with rhizome δ^34^S (R^2^ = 0.13, p = 0.04). Rhizome [Mn] was not significantly different between locations (F_2,31_ = 2.2, p = 0.13). Rhizome [Cr] were very low across Cockburn and Warnbro Sounds, despite the relatively high sediment [Cr]. Rhizome [Cr] was only above detectable limits at GI 3 m, GI 5 m, GI 7 m, and WS 5 m. Rhizome [Cd] was significantly higher at Warnbro Sound sites (F_2,31_ = 9.0, p = 0.0008), despite low sediment [Cd] across the entire study area.

**Table 4:** Mean metal concentrations in rhizomes of seagrasses growing in Cockburn/Warnbro Sound. Means are calculated from 3 replicates, with standard errors provided in brackets.

Site	Rhizome [Zn] (ppm)	Rhizome [Fe] (ppm)	Rhizome [Mn](ppm)	Rhizome [Cd] (ppm)	Rhizome [Cr] (ppm)
GI 2 m	177.97 (14.6)	23.90 (4.3)	1.60 (0.1)	0.16 (0.007)	0.00 (0)
GI 3 m	155.20 (24.0)	35.63 (12.4)	8.63 (6.3)	0.25 (0.02)	0.07 (0.07)
GI 5 m	88.57 (8.1)	42.50 (4.1)	0.97 (0.2)	0.14 (0.009)	0.17 (0.09)
GI 7 m	168.47 (16.3)	100.53 (27.7)	1.83 (0.1)	0.24 (0.03)	0.30 (0.1)
EB 2 m	134.73 (19.7)	56.03 (14.3)	1.87 (0.7)	0.24 (0.03)	0.00
EB 7 m	79.93 (11.0)	91.47 (19.7)	1.17 (0.5)	0.21 (0.03)	0.00
EB 5 m	100.23 (10.4)	94.97 (16.8)	1.10 (0.2)	0.19 (0.02)	0.00
EB 3 m	49.63 (18.1)	62.00 (22.5)	0.87 (0.3)	0.16 (0.01)	0.03 (0.03)
WS 2 m	124.37 (6.7)	20.27 (2.8)	0.97 (0.03)	0.25 (0.01)	0.00
WS 3 m	23.40*	11.10*	0.60*	0.46*	0.00*
WS 5 m	77.23 (20.6)	86.40 (13.5)	2.40 (0.53)	0.29 (0.07)	0.17 (0.12)
WS 7 m	106.47 (9.4)	58.30 (13.6)	1.17 (0.07)	0.32 (0.04)	0.00
*Cockburn Sound 1977* ^1^	*58.58*	*89.53*	*N/A*	*0.45*	*N/A*
*Mediterranean Sea* ^2^	*44.10*	*170.40*	*8.50*	*1.12*	*0.54*

*Root biomass was too low at WS 3 m for root S and δ^34^S in one replicate, ^1^Talbot and Chegwidden (1982); ^2^Tovar-Sanchez *et al*. (2010)

**Figure 3:**
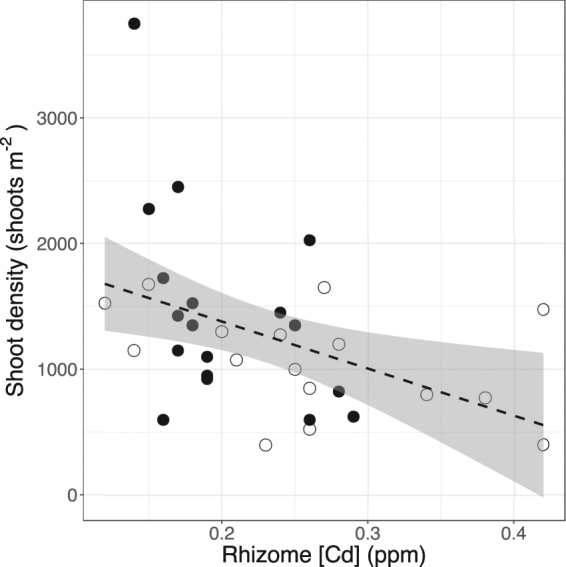
Scatterplots showing relationship rhizome cadmium concentrations and shoot densities. Dashed line represents linear model, grey outline shows 95% confidence interval. White dots denote replicates from sites with a history of seagrass declines, while black dots denote replicates from sites with steady seagrass trends^12^.

Rhizome [Cd] was significantly negatively correlated to seagrass shoot density (R^2^ = 0.20, p = 0.005, n = 36, Fig. 3), leaf biomass (R^2^ = 0.27, p = 0.002, n = 36), rhizome biomass (R^2^ = 0.22, p = 0.003, n = 36), and root biomass (R^2^ = 0.17, p = 0.008, n = 35). No other metals were correlated with changes in seagrass biomass, shoot density, or productivity (data not shown).

Rhizome [Cd] and [Fe] were typically lower than comparable rhizome metal concentrations measured in Cockburn Sound in 1977^29^, and rhizome [Cd] from Mediterranean *P. oceanica* meadows^30^, while Rhizome [Zn] were higher. No comparable historical rhizome [Cr] or [Mn] data from Cockburn Sound is available, but concentrations in were lower than Mediterranean *P. oceanica* rhizomes^30^.

## Discussion

Seagrasses in Warnbro Sound and at some locations in Cockburn Sound have tissues with depleted δ^34^S relative to nearby sites and consistent with sulfide intrusion. Seagrass δ^34^S was significantly depleted in leaves, rhizomes and roots at sites with recorded trends of reductions in shoot densities. Moreover, δ^34^S values at impacted sites were well below local, regional and global averages^17,21^, suggesting that these seagrasses are impacted by sediment stressors that could be contributing to the trend of declining shoot densities^31^. Seagrass management plans in Cockburn and Warnbro Sound focus exclusively on characterising the health of the ecosystem using above-ground seagrass metrics (e.g. shoot density) that have a cause-effect pathway directly related to increased light attenuation associated with historically poor water quality^32^. This approach discounts the role that sediment health has on the growth and mortality of seagrasses^8,33^. Though water quality caused the initial, widespread loss of seagrass in Cockburn Sound in the 1960s–1980s, recent declines are not related to water quality^11^. However, spatial patterns of seagrass loss are best explained by depleted δ^34^S; an indicator of sulfide intrusion that is a stressor that the existing seagrass management plans would not be able to address^32,34^.

The susceptibility of seagrasses to sulfide intrusion is governed by a variety of environmental factors. We expected sedimentary organic matter (OM) to be correlated to sulfide intrusion, but found no relationship between OM and leaf, rhizome or root δ^34^S. We predicted this relationship based on increased OM increasing sulfate reduction rates in sediments, a trend that has been observed in previous seagrass sediments enriched with OM^13^. However, other studies have shown that sulfide intrusion is not always related to sediment organic matter content^16^. The impact of organic loading on sulfide intrusion is likely dependent on a complex set of variable including the chemical composition of OM, microbial community functional traits, and the ability of the seagrasses to oxygenate their rhizosphere^4,35^. In addition, increases in water temperature could increase sulfide intrusion into seagrasses by enhancing sulfate reduction rates in sediments^36^ while simultaneously reducing the capacity of the plant to oxygenate the surrounding rhizosphere by increasing metabolic/respiration rates. Sulfide intrusion may become a greater driver of seagrass loss as temperatures increase from climate change, especially during extreme temperature events such as marine heatwaves^37^. Differences in seagrass δ^34^S could also be partially explained by differences in sediment δ^34^S across the study area^17^ that was not measured in this experiment. Previous studies have found acid-volatile sediment δ^34^S to be relatively stable in *P. sinuosa* meadows in Cockburn Sound^17^. However, sediment sulfide pools and δ^34^S can change appreciably over diel and seasonal cycles^38,39^. As such, future research focusing on drivers of sediment sulfide concentrations and intrusion into seagrass tissues in Cockburn and Warnbro Sounds over diel and seasonal time scales would provide useful information, with a particular focus on measuring nocturnal sulfide dynamics, given this is the period when seagrasses are more vulnerable to sulfide intrusion given there is no photosynthetic oxygen production and oxygen balance in seagrass tissues is driven by water column oxygen concentrations and current speeds^40^. In addition, future research could also explicitly examine any physiological damage that increased sulfide intrusion has on *P. sinuosa* and contribute to the development of a sediment-stress functional indicator for use in these meadows, similar to that developed in *Halophila ovalis* meadows^41^, which would aid in management efforts.

Sediment Fe was positively correlated to root and rhizome δ^34^S values, confirming our third hypothesis, and indicating that sediment Fe provides some protection against sulfide intrusion. Even small increases in sediment Fe can lessen sulfide intrusion by decreasing dissolved sulfide pools in sediments, particularly in calcium carbonate sediments with naturally low sediment Fe like in Cockburn Sound^42^. The subsequent role of sediment Fe would depend on reoxidation of any FeS products in order to maintain buffering capacity against a build-up of free sulfides in sediments^5^. Reoxidation of sediments in Cockburn Sound is relatively high, leading to low sulfate reduction rates and ensuring the buffering capacity of sedimentary Fe^17^.

Sediment Fe is currently 1–2 orders of magnitude lower in present day Cockburn Sound sediments compared to those measured in 1977^43^. This difference may partially be explained by different extraction techniques; with previous studies extracting metals from sediments using an HCl/HNO_3_ extraction^43^ instead of the ethylenediaminetetraacetic acid (EDTA) extraction used in this study. EDTA extractions are considered more efficient in extracting some metals from sulfate-rich marine sediments than HCl^44^, and provides a more accurate estimate of metal bioavailability^45^. However, both techniques would differ in the pools of Fe species that they extract, with dilute HCl extractions targeting Fe(OH)_3_, FeCO_3_ and FeS, while EDTA extractions would target Fe(OH)_3_, FeOOH and Fe_2_O_3_^46^. As such, the type of extractions used may contribute to differences measured between the two studies, with differences of ~20% previously observed in marine sediments^45^. In addition, a reduction of industrial inputs into the Sound, especially the loss of the iron refinery and steel mill, across this time period may also contribute to a reduction in sedimentary Fe. If so, the tighter regulation of coastal industry along Cockburn Sound may have concurrently improved growing conditions (by improving water quality) but left seagrass more vulnerable to sediment stressors, and contribute towards the lack of widespread seagrass recovery in the corresponding time period. Sediment Fe should be included in a suite a parameters to determine the sites where seagrasses are more vulnerable to sulfide intrusion, and addition of Fe to sediments could be used to alleviate sulfide stress for seagrasses in impacted areas or prior to restoration attempts^19,42,47^. In addition, further research into sediment Fe using sequential extraction techniques would provide the most comprehensive approach to assessing the mobility of Fe in sediments across the area, and the capacity for sediment Fe to act as a buffer against accumulation of sedimentary sulfides and subsequent intrusion into seagrass tissues.

Heavy metals were historically released into Cockburn Sound from surrounding industrial activity and are a concern as they have limited mobility in the environment^48^ and have known negative impacts on seagrasses^22,49^. We found lower concentrations of all metals in sediments relative to previous surveys in Cockburn Sound^43^. These differences again may result from different extraction techniques, or different metal speciation in sediments, which more detailed sequential extractions could reveal^44^. Sediment and seagrass metal concentrations did not generally show clear patterns at sites with and without seagrass declines, suggesting that heavy metals from industrial inputs are not contributing to the long-term declines of seagrass in Cockburn Sound and surrounding areas. One exception to this was seagrass Cd concentrations, which were elevated in Warnbro Sound sites and negatively correlated with seagrass biomass and shoot density (Fig. 3). Seagrasses have previously been used as bioindicators of environmental Cd concentrations^50^, and previous experiments have showed a limited effect of Cd uptake on leaf photosynthesis^22^ and accumulation in leaves of *Halophila stipulacea* ^51^. However, we are not aware of studies examining other physiological impacts on increased Cd uptake on seagrasses, including effects on sulfur metabolism. Increased Cd uptake in terrestrial angiosperms interferes with sulfur metabolism by increasing the activity of enzymes and expression of genes related to reductive sulfate assimilation, thereby increasing sulfide uptake through roots^52^. Cadmium uptake could play a similar role in seagrasses, and lead to an increase in vulnerability to sulfide intrusion.

Seagrass zinc concentrations were the only heavy metal concentrations to increase in seagrass rhizome relative to concentrations previously measured^29^, despite corresponding decreases in sediment Zn^43^. Seagrass Zn was particularly high at Garden Island sites (Table 4). Offshore sites in Cockburn Sound have previously been hypothesised to have higher Zn concentrations in sediments caused by fine contaminated sediments from industrial development accumulating offshore^53^. There was no strong correlation between Zn and shoot density or biomass, likely because Zn is an essential micronutrient for seagrasses^23^, with toxic effects only encountered in contaminated waters with high Zn concentrations (e.g. 570 µg l^−1^ ^22^). However, seagrass tissues enriched in Zn can be transferred to associated invertebrates leading to bioaccumulation, particularly in detritivores and herbivores that directly consume dead or living seagrass tissues^54^. Metal concentrations were previously measured in sediment associated invertebrates^29^, and would provide a baseline for comparison for future studies of bioaccumulation of Zn in seagrass-associated invertebrates in Cockburn Sound.

Kendrick *et al*.^3^ hypothesised that the environment in Cockburn Sound had been “altered to an environment not suited to large-scale recolonization by Posidonia species”. Our data suggests that sulfide stress from sediments is contributing to this observed lack of seagrass recolonization (in spite of improved water quality), resulting in a phase shift in the benthic community of Cockburn Sound. Such phase shifts may be common after seagrass declines, particularly when declines have enriched sediments in OM from dead seagrass tissues. These organic matter inputs would alter oxygen and nutrient availability in sediments, potentially resulting in ‘legacy problems’ that prevent seagrass recovery even after initial drivers of loss are mediated. The loss of oxygen inputs from seagrass roots would also lead to a shift in the balance of benthic metabolism, favouring anaerobic respiration and more sulfidic conditions. We suggest that there is a need to integrate above- and below-ground measurements that include indicators in seagrasses, sediments, and the water column in seagrass management programs to provide a comprehensive assessment of seagrass health and potential pressures, even where water quality appears to be the sole driver of initial seagrass declines.

## Methods

### Study Sites and experimental design

**Figure 4:**
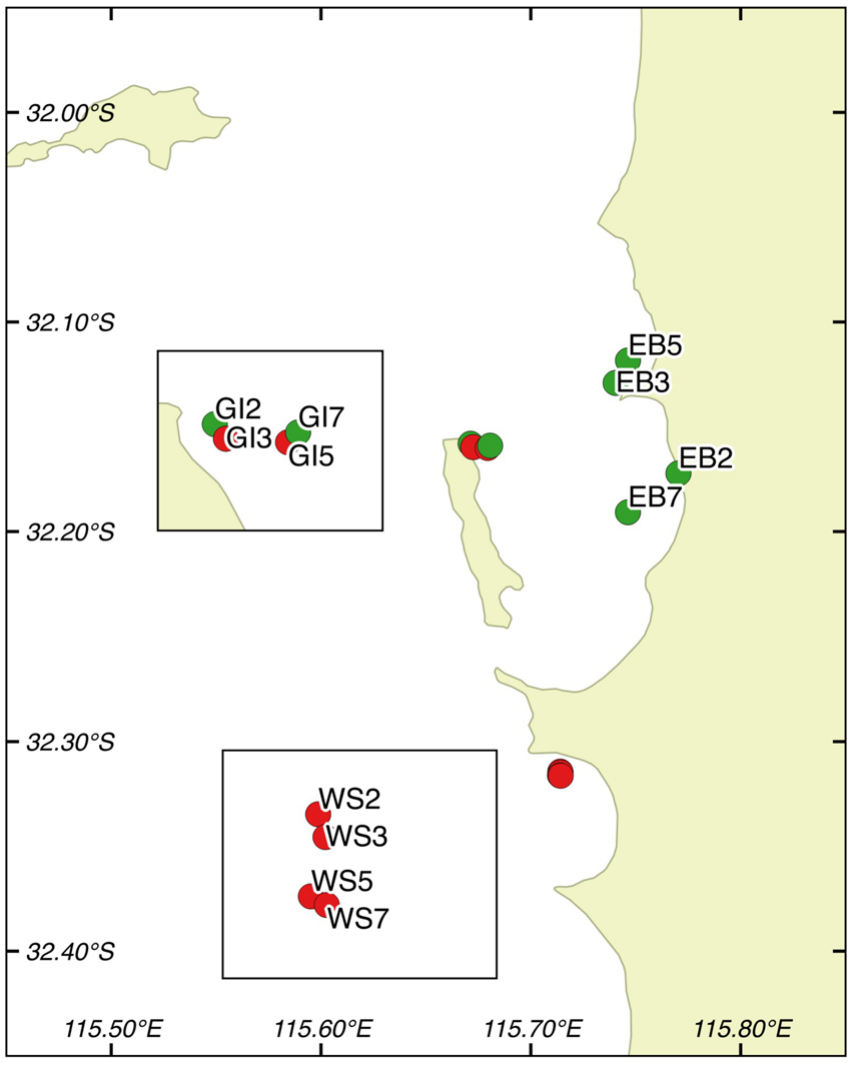
Map showing study area. GI = Garden Island, WS = Warnbro Sound, EB = Eastern Banks. Red icons denote sites with a history of seagrass declines, while green icons represent sites with steady shoot density trends^12^. Map was created with QGIS version 2.8.2 (Open Source Geospatial Foundation Project, http://qgis.osgeo.org).

Three locations (each with four sites) were sampled between March-May 2015: Eastern Banks (EB), Garden Island (GI), and Warnbro Sound (WS) (Fig. 4). At each location, sites were stratified by depth (2 m, 3 m, 5 m, and 7 m). Sites sampled had varying degrees of shoot declines based on historical monitoring data from the annual Cockburn Sound Management Council monitoring project^12^. Six sites (WS 2, 3, 5, 7 m; GI 3 and 5 m) had highly significant shoot declines, while the remaining six sites had no record of shoot declines (GI 2 and7m, EB 2, 3, 5, 7 m). *Posidonia sinuosa* was the dominant seagrass species at all sites.

Within each site, three 20 cm × 20 cm quadrats were set up. During the initial visit to each site, all *P. sinuosa* shoots within each quadrat were marked for productivity using a standard hole punching technique^55^. Sites were then revisited four weeks later, where quadrats with hole punched seagrasses were completely extracted using a 25-cm corer. Seagrasses were bagged, stored on ice and transported in dark to the laboratory for analysis. In addition, approximately 50 ml of sediment adjacent to each quadrat (surficial sediment to 10 cm) was collected using a 50 ml syringe with a cut-top. Sediment samples were transferred to 50 ml plastic tubes, stored on ice, and transported to the laboratory for analysis.

### Seagrass biomass and productivity

Numbers of shoots within each quadrat (after extraction) were counted to determine shoot density. Adherent epiphytes and sediments were removed from seagrass leaves by gently scraping with a razor blade. Seagrasses were then sorted into leaves, rhizomes, and roots, dried at 60 °C and weighed for biomass. Seagrass productivity was measured using a standard technique^55^ that involved measuring the distance of the hole-punch above leaf sheaths on all seagrass leaves and weighing newly produced biomass after drying at 60 °C. This value was then averaged over 1 month to provide a productivity rate in mg sh day^−1^. Dried seagrass tissues were then ground finely using a ball mill for sulfur (S) analysis, or mortar and pestle for metal analysis.

### Seagrass total sulfur and δ^34^S isotope ratios

Ground leaf, root, and rhizome samples were weighed into tin capsules together with vanadium pentoxide and analyzed by elemental analyzer combustion continuous flow isotope ratio mass spectroscopy (EA-C-CF-IRMS) at Iso-analytical, United Kingdom. The stable isotopic signatures were reported in standard delta notation (units per mill, ‰) as:

1 $${{\rm{\delta }}}^{34}{\rm{S}}=[({{\rm{R}}}_{{\rm{sample}}}/{{\rm{R}}}_{{\rm{standard}}})-1]\times 1000$$

where R = ^34^S/^32^S. The international standard for δ^34^S is the Canyon Diablo Troilite (CDT) a meteorite of FeS used as a standard zero point for expression of sulfur isotopes. Average δ^34^S values can provide an indication of the relative intrusion of sulfides from sediments compared to sulfates from the water column. More negative δ^34^S values indicate a higher contribution of sulfides from sediments (sediment average: −15‰ to −25‰), while a more positive value indicates that sulfate from the water column is dominant S source for the plant (seawater average: +21‰)^21^.

### Sediment characteristics and heavy metal analysis

Organic matter content of sediments was determined by the difference in sediment dry weight after combustion of samples at 550 °C for 8 hours^56^. Elemental analysis of sediment and seagrass rhizome extracts (Cd, Cr, Fe, Mn, Zn for seagrass, Cd, Cr, Fe, Mn, Zn, Cu for sediment) were performed on a PerkinElmer optima 5300DV inductively couple plasma optical emission spectrometer (ICP-OES) at the University of Western Australia. Sediment extracts were prepared by shaking 5 g of dried sediment with 45 ml 0.05 M EDTA solution^44^. EDTA is commonly used as an extractant of sediment metals, and is assumed to extract metals on exchange sites of both inorganic and organic complexes, providing a good representation of metals that are bioavailable^44,45^. Seagrass extracts were prepared by digesting 0.3 g of seagrass rhizome using a conc. HNO_3_/H_2_O_2_ digest^57^.

### Statistical analysis

Linear regressions were used to determine relationships between seagrass biomass, seagrass S and δ^34^S content and seagrass metal concentrations. In addition, linear regression was used to investigate relationships between sediment OM/metal content, and seagrass biomass and seagrass elemental content. Differences in sediment and seagrass parameters between sites and depths were investigated using two-way ANOVAs. Data were square-root transformed where assumptions of heterogeneity were not met. All statistical analysis was performed using R version 3.2.2^58^ and graphs produced using ggplot^59^.

### Data accessibility

Data in this article are published onthe FigShare data Repository at https://figshare.com/s/3e705529e7c311830c33 (doi:10.6084/m9.figshare.5103340).
